# Editorial on special issue on “Immunobiology of Viral Infections”

**DOI:** 10.1007/s00430-023-00761-0

**Published:** 2023-03-30

**Authors:** Hanna-Mari Baldauf, Asisa Volz

**Affiliations:** 1 Max von Pettenkofer Institute & Gene Center, Virology, National Reference Center for Retroviruses, Faculty of Medicine, LMU München, Munich, Germany; 2 Institute of Virology, University of Veterinary Medicine Hannover, Foundation, Hanover, Germany; 3 German Center for Infection Research, partner site Hanover-Braunschweig, Hanover, Germany

A host cell has many ways how to interfere with invading viral pathogens. The fastest reaction is provided by the innate immunity, but also the adaptive immunity plays an important role to control viral replication. In the first case, viruses can be recognized by pattern recognition receptors (PRRs) or cytosolic sensors, which then trigger production and release of interferons (IFNs) and upregulation of IFN-stimulated genes, which further block viral replication by distinct mechanisms. For efficient replication, viruses, however, have evolved counteracting mechanisms. Thus, those virus-host interactions culminate in an arms-race with the host, which also leave genetic imprints on both, the viral genes and the antiviral host genes. From the viral point of view, it is of course necessary to overcome limitations imposed by the host. The aim of this special issue is to provide the readers the latest findings concerning the immunobiology of viral infections.

In this special issue, Lee and colleagues summarize recent insight how severe acute respiratory syndrome coronavirus 2 (SARS-CoV-2) has evolved different mechanisms to evade IFN induction and signaling [1]. SARS-CoV-2 is mostly recognized by RIG-I-like receptors (RLRs) and Toll-like receptors (TLRs) 2 and 4, by a yet unknown pattern recognition. To avoid the host’s innate immune response, Lee et al. nicely summarize the functions of all non-structural proteins that exert counteracting mechanisms. For example, Nsp14 prevents recognition of SARS-CoV-2 mRNAs and inhibits binding of IFN to the cell. But not only non-structural proteins exert antagonizing activities. Both structural (e.g., nucleocapsid of SARS-CoV-2) and accessory proteins, such as proteins from the ORF3 and ORF7 locus and ORF9b, help the virus to not activate the host’s innate immune responses, yet the underlying mechanisms still need to be further explored.

Once, IFN is able to mount an antiviral response, IFN-stimulated genes, such as serine incorporator proteins (SERINC, SER) and guanylate binding proteins (GBPs), have evolved additional mechanisms to counteract viral infections. Cano-Ortiz and colleagues described in detail newest insight in the evolution and expression of SERINC proteins. Specifically, they focused on SER3 and SER5, which inhibit fusion pore formation during entry of human immunodeficiency virus (HIV) into target cells [2]. Next to HIV, amphotropic murine leukemia virus (MLV) and influenza A virus have also been shown to be restricted by SER5. Retroviruses, such as HIV, MLV and equine infectious anemia virus (EIAV), have evolved countermechanisms and code for viral proteins that are able to interfere with SER’s antiviral activity. Moreover, both SER3 and SER5 have also been described to have an additional role beyond antiviral activity in innate sensing and signaling. Another antiviral protein family is GBPs. In this issue, Schelle, Côrte-Real and colleagues give valuable insight in recent advances in the field of GBPs [3]. They summarize current knowledge on the structure, dimerization and polymerization, localization and evolution on one hand as well as GBPs’ function in innate immunity on the other hand. Here, they specifically focus on the functions of GBPs in plants, invertebrates and vertebrates since human GBPs have recently gained more attention. Yet, detailed understanding of the role of GBPs in the species-specific context is still lacking, which would provide a more profound understanding on the complex GBP network and their functions.

The adaptive immunity does not provide the first line of defense, but plays also an important part in the control of viral infections. Cytomegaloviruses (CMV) are masters of controlling both the innate and the adaptive immune responses. As reviewed by Hamdan et al., cytomegaloviruses are controlled by the immune system, but are still able to establish latency [4]. Reactivation of the virus poses a problem, especially in immunocompromised hosts. Recognition of infected cells is CD8 T cell-driven, yet CMV encodes for viral regulators of antigen presentation (vRAPs) that control trafficking of pMHC-I complexes to the cell surface. More important, the avidity of the CD8 T effector cells (TEC) determines whether viral infections are being recognized or not.

In contrast to innate immune responses, adaptive immune responses and pathogenesis of viruses are usually studied in vivo. At the beginning of the SARS-CoV-2 pandemic, no animal model was readily available. Clever & Volz describe how different animal models have been tested for their suitability to serve as an animal model for SARS-CoV-2, outlining the advantages and disadvantages of each model system [5]. They further highlight how the identification of ACE2 as entry receptor boosted the development of different ACE-2 mouse models. Here, they discuss the different mouse models, such as the K18-hACE2 mice being used for SARS-CoV-2 infection and vaccination studies. Nevertheless, they mentioned how important the selection of the right animal model is to get as close as possible to the situation in humans.

As a final report in this issue, Kolb and colleagues have summarized recent findings how immune complexes determine the disease outcome of COVID-19 [6]. IgG subclass antibodies are produced by B cells a few days after infection. They bind to their respective antigens, forming thereby IgG immune complexes (Ig-ICs), which usually get cleared by phagocytic cells. If not, they circulate in the host as soluble immune complexes (sIGs) and can have a negative impact on disease severity. Thus, clearance of those sIGs by different means, such as plasmapheresis or application of non-human IgGs (IVIg), might improve the clinical outcome of COVID-19.

## Data Availability

No data was generated for this editorial.
